# Repercussões da Pandemia de COVID-19 na Prática Assistencial de um Hospital Terciário

**DOI:** 10.36660/abc.20200436

**Published:** 2020-09-17

**Authors:** André Luiz Cerqueira Almeida, Thyago Monteiro do Espírito Santo, Maurício Silva Santana Mello, Alexandre Viana Cedro, Nilson Lima Lopes, Ana Paloma Martins Rocha Ribeiro, João Gustavo Cerqueira Mota, Rodrigo Serapião Mendes, Paulo André Abreu Almeida, Murilo Araújo Ferreira, Diego Moreira Arruda, Adriana Aguiar Pepe Santos, Vinícius Guedes Rios, Maria Rosa Nascimento Dantas, Viviane Almeida Silva, Marcos Gomes da Silva, Patrick Harrison Santana Sampaio, André Raimundo Guimarães, Edval Gomes Santos

**Affiliations:** 1 Santa Casa de Misericórdia de Feira de Santana Feira de SantanaBA Brasil Santa Casa de Misericórdia de Feira de Santana, Feira de Santana, BA - Brasil; 2 Escola de Ecocardiografia da Bahia Feira de SantanaBA Brasil Escola de Ecocardiografia da Bahia, Feira de Santana, BA - Brasil; 3 Instituto Nobre de Cardiologia Feira de SantanaBA Brasil Instituto Nobre de Cardiologia,Feira de Santana, BA - Brasil; 4 Universidade Estadual de Feira de Santana Feira de SantanaBA Brasil Universidade Estadual de Feira de Santana, Feira de Santana, BA - Brasil; 5 UNIFACS SalvadorBA Brasil UNIFACS Curso de Medicina, Salvador, BA - Brasil

**Keywords:** COVID-19, Pandemia, Coronavirus, Betacoronavírus, Oncologia, Hospitalização, Serviços Médicos de Emergência

## Abstract

**Fundamento:**

Ainda não temos informações acerca do impacto da pandemia da COVID-19 sobre a atividade médica assistencial no Brasil.

**Objetivo:**

Descrever as repercussões da pandemia da COVID-19 na rotina de atendimentos em um hospital terciário, referência regional em cardiologia e oncologia.

**Métodos:**

Estudo de corte transversal. Foi realizado levantamento dos atendimentos no período de 23/03/2020 (fechamento do comércio local) até 23/04/2020 (P20) e comparado com o mesmo período em 2019 (P19).Resultados: Detectamos redução no número de consultas cardiológicas, teste ergométrico, Holter, monitorização ambulatorial da pressão arterial, eletrocardiograma e ecocardiograma (90%, 84%, 94%, 92%, 94% e 81%, respectivamente). Em relação à cirurgia cardíaca e cateterismo cardíaco, houve redução de 48% e 60%, respectivamente. Aumento no número de angioplastia transluminal coronária (33%) e de implante de marca-passo definitivo (29%). Houve 97 internamentos na UTI em P19, contra 78 em P20, redução de 20%. Diminuição dos atendimentos no pronto-socorro cardiológico (45%) e nos internamentos na enfermaria de cardiologia (36%). Houve diminuição nas consultas oncológicas de 30%. Sessões de quimioterapia reduziram de 1.944 para 1.066 (45%). Sessões de radioterapia diminuíram 19%.

**Conclusão:**

A COVID-19 provocou redução considerável no número de consultas nos ambulatórios de cardiologia, oncologia e demais especialidades. Houve uma preocupante diminuição no número de cirurgias cardíacas e nas sessões de quimioterapia e radioterapia nas semanas iniciais da pandemia. A procura por atendimento no pronto-socorro cardiológico, assim como as internações na UTI e enfermaria cardiológicas, também reduziram, gerando preocupação acerca da evolução e prognóstico destes pacientes portadores de outras patologias, que não a COVID-19, nestes tempos de pandemia. (Arq Bras Cardiol. 2020; [online].ahead print, PP.0-0)

## Introdução

Em dezembro de 2019, foram descritos os primeiros casos de indivíduos infectados pelo novo coronavírus (SARS-COV 2) em Wuhan, China, que rapidamente alastrou-se pelo país, levando a grande número de mortes e de hospitalizações.^1^ Em curto período de tempo, a doença pelo coronavirus 2019 (COVID-19) ultrapassou os limites da China e alcançou outros países da Ásia, Europa e Américas. Até meados do ano 2020 cerca de 8,5 milhões de pessoas tinham testado positivo para o novo coronavirus em mais de 187 países e 200 territórios, sendo 960 mil testes positivos no Brasil. Na mesma época, registrava-se próximo de 450 mil mortes no mundo atribuídas à COVID-19 (47 mil no Brasil).^2^ O primeiro caso de COVID-19 no Brasil foi confirmado em São Paulo, em 26/02/2020.^3^ Em 06/03/2020, a Secretaria da Saúde do Estado da Bahia confirmou o diagnóstico do 1º caso da COVID-19 na Bahia, mais precisamente na cidade de Feira de Santana. Tratava-se de uma mulher de 34 anos, que retornou da Itália em 25 de fevereiro, com passagens por Milão e Roma, onde aconteceu a contaminação. Em 11 de março de 2020, a Organização Mundial da Saúde classificou a COVID-19 como uma pandemia, sendo que em 20 de março de 2020 o Ministério da Saúde do Brasil declarou, em todo o território nacional, estado de transmissão comunitária da doença.^4^ Isso significa que o vírus já circulava em todo o país. Na mesma data, o gestor público local de Feira de Santana emitiu um decreto normativo decretando o fechamento de todo o comércio varejista e atacado, no âmbito do município, a partir do dia 23/03/2020.

Investigações científicas estão em andamento em todo o mundo e, por se tratar de um evento ainda novo, muitos esforços estão sendo despendidos para respaldar os profissionais de saúde e os gestores. No Brasil, em particular, toda e qualquer condição sanitária precisa considerar a dimensão territorial e as diferenças regionais do país. Durante uma pandemia como a atual, o formato de gestão tripartite do Sistema Único de Saúde (SUS) ganha importância, visto que as decisões devem ser compartilhadas entre o governo federal, os estados e os municípios.

Durante a pandemia, alguns autores registraram mudanças na estrutura do sistema de saúde em países da Europa e nos EUA, com importante declínio no número de atendimentos e procedimentos médicos não associados à COVID-19, incluindo aqueles de alta complexidade.^5 - 12^ Estas mudanças podem gerar, como efeito colateral, atraso diagnóstico e/ou terapêutico e consequente aumento no risco de descompensação de doenças crônicas.

Ainda não temos informações acerca do impacto desta pandemia, e subsequentes ações governamentais, sobre a atividade médica assistencial no Brasil. O objetivo deste trabalho foi descrever as repercussões da pandemia da COVID-19 sobre o número de consultas, exames, internações e procedimentos médicos realizados em um hospital terciário, referência regional em cardiologia e oncologia.

## Métodos

Estudo de corte transversal realizado em um hospital terciário com 110 leitos de enfermaria e 12 leitos de UTI. Trata-se de uma unidade referência regional em cardiologia (cirurgia cardíaca, cateterismo, angioplastia, dispositivos eletrônicos implantáveis, ecocardiograma e pronto-socorro [PS] cardiológico) e oncologia (quimioterapia, radioterapia e cirurgias oncológicas), que presta atendimento a pacientes tanto do SUS quanto do sistema de saúde suplementar. Para analisarmos a repercussão da pandemia da COVID-19 sobre as práticas assistenciais do hospital, foi feito um levantamento do número de atendimentos nos vários setores da unidade no período de 23/03/2020 (data do fechamento do comércio local) até 23/04/2020 (P20) e comparamos com os atendimentos realizados no mesmo período do ano de 2019 (P19). Trata-se de uma amostra de conveniência, com a inclusão de todos os pacientes com dados disponíveis em registros eletrônicos nos períodos supracitados. Foram coletados dados acerca das práticas assistenciais e admissões nos seguintes setores do hospital: UTI (predominantemente cardiológica), cirurgia cardíaca, cirurgia não cardíaca, cateterismo cardíaco, angioplastia transluminal coronária (ATC), implante de marca-passo, consulta em cardiologia, ecocardiografia, monitorização ambulatorial da pressão arterial (MAPA), Holter, eletrocardiograma (ECG), teste ergométrico, PS cardiológico, internamento na unidade de cardiologia, laboratório de análises clínicas, consulta em oncologia, sessões de quimioterapia, sessões de radioterapia, ultrassonografia, tomografia computadorizada, endoscopia, colonoscopia e retossigmoidoscopia.

### Análise Estatística

Realizamos uma análise descritiva dos dados obtidos na amostra. As variáveis nominais ou categóricas foram descritas por seus valores absolutos. As diferenças de eventos observada entre os dois períodos investigados foram descritas pelas razões absolutas e relativas. A análise dos dados, assim como a construção dos gráficos, foi feita com o auxílio do Excel®, Microsoft 365®.

### Aspectos Éticos

Este estudo foi aprovado pelo Comitê de Ética em Pesquisa da Universidade Estadual de Feira de Santana sob o número de protocolo CAAE: 31056220.0.0000.0053. Todos os procedimentos envolvidos nesse estudo estão de acordo com a Declaração de Helsinki de 1975, atualizada em 2013. O levantamento foi realizado através da pesquisa direta dos registros do hospital, após autorização expressa da instituição hospitalar. O pesquisador responsável assinou um termo de compromisso para utilização dos dados dos registros do hospital.

## Resultados

**Figura 1:**
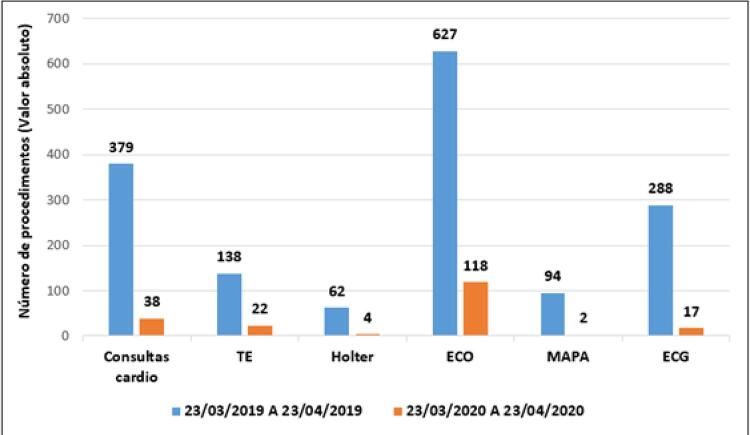
– Número de consultas e exames em cardiologia. Cardio: cardiologia; TE: teste ergométrico; ECO: ecocardiograma; MAPA: monitoramento ambulatrorial da pressão arterial; ECG: eletrocardiograma.

No P19, o ambulatório de cardiologia realizou 379 consultas, sendo que este número caiu para 38 no P20, com uma redução de 90% ( Figura 1 ). Observou-se ainda uma redução no número de teste ergométrico (84%), Holter (94%), MAPA (92%) e ECG (94%). Em relação ao ecocardiograma, foram 509 exames a menos em P20, o que corresponde a uma redução de 81% ( Figura 1 ). A redução foi de 470 ecocardiogramas ambulatoriais (88%) e de 39 exames realizados em pacientes internados no hospital (41%).

**Figura 2:**
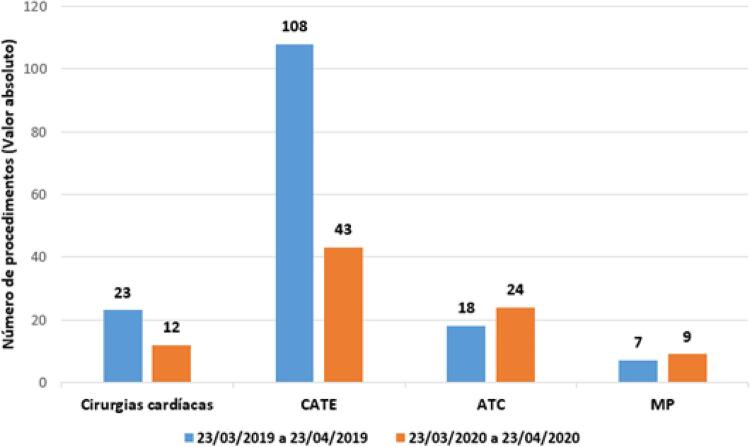
– Movimento no setor de cardiologia intervencionista. CATE: cateterismo cardíaco; ATC: angioplastia transluminal coronária; MP: marcapasso.

Em relação à cirurgia cardíaca e cateterismo cardíaco, a redução foi de 48% e 60%, respectivamente ( Figura 2 ).

Houve um aumento no número de ATC na ordem de 33% e de implante de marca-passo definitivo (29%) ( Figura 2 ).

**Figura 3:**
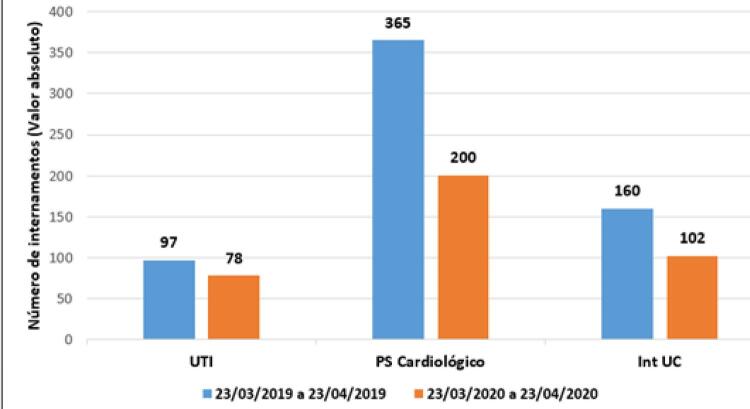
– Número de internamentos na unidade de cardiologia. PS: pronto socorro; Int CV: internamento na unidade de cardiologia.

Noventa e sete pacientes foram internados na UTI em P19, contra 78 em P20, redução de 20%. A redução também foi observada nos atendimentos realizados no PS cardiológico (45%) e nos internamentos na enfermaria de cardiologia do hospital (36%) ( Figura 3 ).

**Figura 4:**
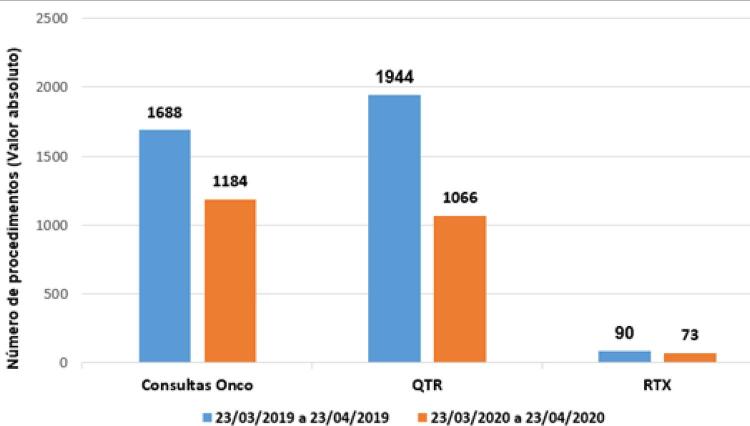
– Movimento no setor da oncologia. Onco: oncologia; QTR: quimioterapia; RTX: radioterapia.

O setor de oncologia também sofreu redução considerável nos atendimentos durante a fase inicial da pandemia da COVID-19. Os oncologistas realizaram 1.688 consultas em P19. Este número caiu para 1.184 consultas em P20, uma redução de 30%. O número de sessões de quimioterapia caiu de 1.944 para 1.066, declínio de 45%. Já as sessões de radioterapia diminuíram 19% ( Figura 4 ).

**Figura 5:**
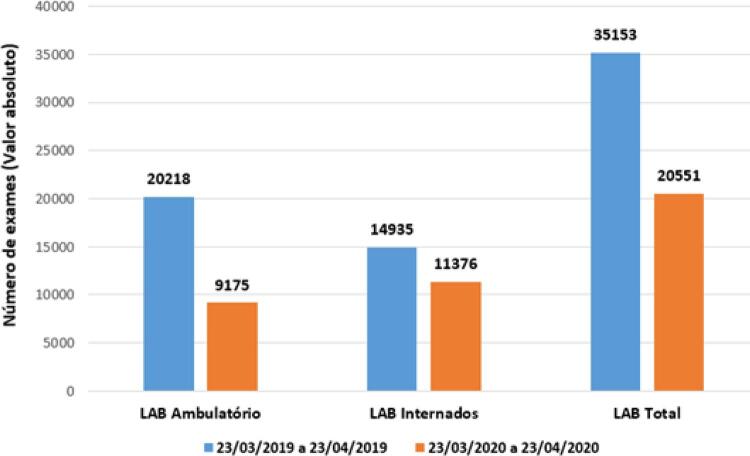
– Movimento do laboratório de análises clínicas. LAB: laboratório.

A redução no número de exames de análises clínicas foi na ordem de 42%. Esta diminuição ocorreu tanto entre os pacientes ambulatoriais quanto entre os internados, sendo que a queda foi maior entre os primeiros ( Figura 5 ). Foram realizadas 337 dosagens da troponina em 2019, contra apenas 59 em 2020, declínio de 82%.

**Figura 6:**
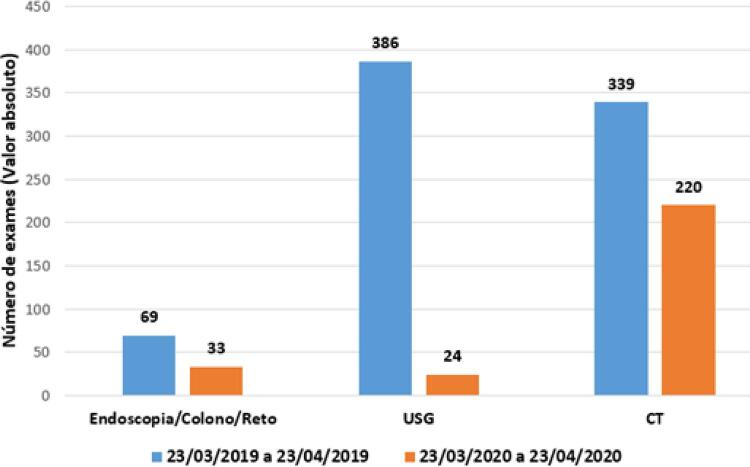
– Movimento nos serviços de exames de imagem. Endoscopia: endoscopia digestiva alta; Reto: retosigmoidoscopia; Colona: colonoscopia; USG: ultrassonografia; CT: tomografia computadorizada.

Houve diminuição também no número de endoscopia/colonoscopia/retossigmoidoscopia (52%), ultrassonografia (94%) e tomografias computadorizada (35%) ( Figura 6 ).

**Figura 7:**
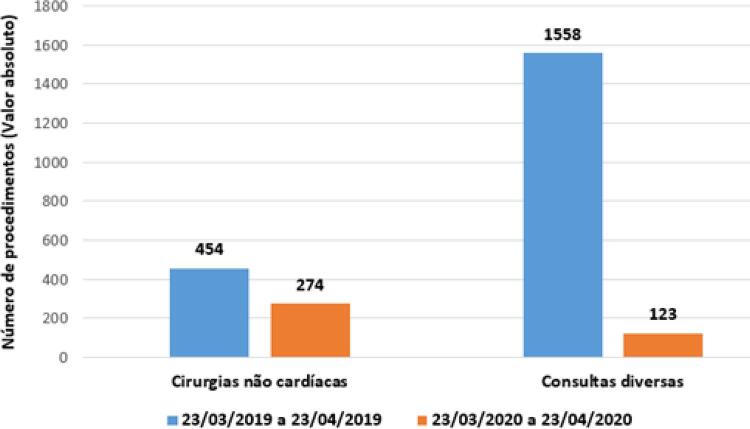
– Número de consultas e cirurgias não cardíacas.

As cirurgias não cardíacas (geral, oncológica, cabeça e pescoço, ortopédica, entre outras) sofreram redução de 40% no primeiro mês em que foi recomendado o distanciamento social devido à pandemia da COVID-19 na região estudada. O número de cirurgias oncológicas ligadas à urologia diminuiu de 82 para 57 (30%). Já as consultas não-cardiológicas e não-oncológicas reduziram 92% ( Figura 7 ).

O hospital onde os dados foram coletados não é um centro de referência para atendimento de pacientes com a COVID-19. Até a conclusão da coleta dos dados nenhum paciente com a COVID-19 havia sido admitido na unidade.

## Discussão

O presente estudo demostrou que a pandemia da COVID-19 provocou uma redução considerável no número de consultas nos ambulatórios de cardiologia, oncologia e demais especialidades em nosso hospital. Além disso, observamos uma preocupante diminuição no número de cirurgias cardíacas, assim como no número de sessões de quimioterapia e de radioterapia nas semanas iniciais da pandemia. Estas reduções podem trazer consequências desastrosas para os pacientes que necessitam desses tratamentos. A procura por atendimento no PS cardiológico, assim como o número de internações na UTI e enfermaria cardiológicas, também sofreu importante declínio, deixando um inquietante questionamento aos cardiologistas e outros profissionais da saúde no primeiro momento: para onde estão indo os pacientes com complicações cardíacas, associadas ou não à pandemia da COVID-19?

Detectamos uma redução de 45% no número de atendimentos no PS de cardiologia do nosso hospital, com 82% de redução na dosagem da troponina. Isto foi acompanhado por um encolhimento nas taxas de admissão na UTI e enfermaria cardiológicas de 20% e 36%, respectivamente. De modo semelhante, Metzler B et al.,^10^ demonstraram uma redução de 39,4% na admissão de pacientes com síndrome coronariana aguda, durante o primeiro mês do surto da COVID-19 na Áustria. Nos EUA, houve um declínio de 38% no número de coronariografias em casos de IAM com supra de ST (IAMCSST) no primeiro mês da pandemia.^8^ Em nossa casuística, observamos uma redução de 60% no número de cateterismo cardíaco e de 48% nas cirurgias cardíacas. Na Espanha, durante a primeira semana da quarentena devido à COVID-19, notou-se uma diminuição de 56% nos procedimentos diagnósticos cardiovasculares, 48% nos procedimentos terapêuticos coronários, 81% nos terapêuticos estruturais (implante percutâneo de válvula aórtica, fechamento de persistência do canal arterial e comunicação interatrial) e 40% nos casos tratados de IAMCSST.^8^ A redução que observamos na procura por atendimento médico por motivos cardiológicos durante a pandemia, fato que se repetiu em outros países, vai de encontro ao que, habitualmente, costumamos ver em períodos de tragédias. É conhecido o aumento considerável na incidência de IAM e AVC após terremotos e tsunamis.^13 , 14^ Talvez como resultado desta redução na procura espontânea por atendimento médico em casos não relacionados à COVID-19, a região da Lombardia, Itália, apresentou um aumento de 58% na ocorrência de parada cardíaca fora do hospital, no período que abrangeu os primeiros 40 dias do surto da COVID-19.^12^ Este achado mostrou forte associação com a incidência cumulativa de COVID-19 na região estudada. Similarmente, dados publicados no Angioplasty.Org relatam um aumento de 800% na incidência de morte súbita ocorrida em domicílio na cidade de Nova York quando era o epicentro da pandemia.^15^ Muitos destes pacientes podem ter evitado ir ao hospital com receio de se infectarem com a COVID-19.^16^

Diferente dos dados obtidos na Espanha,^8^ observamos um aumento de 33% na realização de ATC. Atribuímos este aumento a uma maior disponibilidade de vagas de UTI do nosso hospital na fase inicial da pandemia da COVID-19. Isto permitiu que pudéssemos realizar o procedimento e encaminhar o paciente para observação na UTI, diferente do que ocorria na fase pré-COVID, quando a UTI estava, invariavelmente, sem disponibilidade de leitos.

A redução que observamos no número de consultas em oncologia (30%), sessões de quimioterapia (45%) e sessões de radioterapia (19%) é um capítulo à parte. Recente estudo, realizado na Inglaterra e Irlanda do Norte, observou que a maioria dos pacientes com câncer, ou suspeitos de terem câncer, não estavam acessando os serviços de saúde durante a pandemia da COVID-19.^17^ Como consequência, estima-se que este surto da COVID-19 tem o potencial de aumentar a mortalidade em cerca 20%, nos próximos 12 meses, nos pacientes com diagnóstico recente de câncer, apenas na Inglaterra.^17^ Além disso, pacientes com câncer têm um risco quase quatro vezes maior de apresentar complicações graves secundárias à COVID-19, comparados aos indivíduos sem câncer.^18^ Portanto, a monitorização dos pacientes oncológicos deve ser redobrada, e não diminuída, durante o curso da pandemia. O desafio de identificar e tratar complicações associadas a alguns quimioterápicos, como miocardite e/ou pneumonite severas, é outra questão a ser enfrentada pelos especialistas em tempos de pandemia da COVID-19.^19^

Os nossos resultados são de grande auxílio para os pacientes, os profissionais de saúde e os gestores hospitalares. Eles trazem à tona um problema grave, que está cursando em paralelo à pandemia da COVID-19: a redução considerável no número de consultas, cirurgias cardíacas e oncológicas, atendimentos em PS cardiológico, cateterismo cardíaco, sessões de quimioterapia e de radioterapia, exames laboratoriais, entre outros procedimentos médicos importantes e necessários para aqueles que não estão com a COVID-19. Uma redução no acesso aos cuidados médicos é associada a um declínio no estado de saúde da população.^20^ O retardo no diagnóstico e tratamento do infarto do miocárdio ou do AVC aumenta o risco de morte.^21^ A interrupção do uso de anti-hipertensivos, mesmo por curtos períodos, pode ocasionar graves complicações cardiovasculares.^22^ A retirada das estatinas aumenta as taxas de eventos em pacientes com síndromes coronárias agudas.^22^ A descompensação não diagnosticada do diabetes tem consequências graves. A demora na realização da cirurgia oncológica pode levar à progressão da doença e piora do prognóstico. O mesmo se aplica em relação às sessões de quimioterapia adjuvantes ou neoadjuvantes. O fato dos pacientes se absterem de ir ao hospital, não significa que as outras doenças desapareceram. Em absoluto! Elas continuam acometendo os pacientes, mas estes não estão procurando o atendimento médico na medida adequada. A questão é: por quê? Entre outros motivos porque, provavelmente, estão com medo de irem ao hospital devido ao risco de contraírem a COVID-19. É possível, ainda, que alguns pacientes com problemas cardiovasculares estejam procurando atendimento médico, mas os sintomas apresentados podem estar sendo confundidos com os da COVID-19. A recomendação expressa para as pessoas ficarem em casa, assim como a restrição na liberdade de trânsito e movimentação, também contribuem para o cenário que detectamos em nosso hospital. Não seria exagero dizer que estes indivíduos também se tornaram vítimas da COVID-19, mesmo sem terem sido acometidos da doença.

Independente da causa, os resultados da nossa pesquisa são um espelho do que está acontecendo em vários outros locais. Tais fatos têm o potencial de gerar um efeito colateral preocupante: um substancial incremento na morbidade e na mortalidade, a curto e médio prazo, por causas outras que não a infecção causada pelo SARS-COV 2.^16^ Esta seria a outra face da tragédia da COVID-19. É possível que, em breve, nos deparemos com uma curva ascendente, pós-pandemia, composta por pacientes com graves complicações secundárias a patologias que poderiam ter sido adequadamente tratadas, caso tivessem sido atendidas previamente e em um momento, teoricamente, mais favorável.

Os nossos resultados abrem perspectivas para que outros pesquisadores investiguem qual o real desfecho destes pacientes que não estão comparecendo às consultas médicas eletivas, às sessões de quimioterapia e radioterapia, ao PS de cardiologia, assim como aqueles que não estão realizando os exames de rotina e/o as cirurgias oncológicas ou cardíacas no período da pandemia da COVID-19. Isto complementaria os nossos achados e traria importantes informações para a comunidade médica e para os próprios pacientes.

Nosso trabalho apresenta algumas limitações. Trata-se de um estudo unicêntrico e o período de observação foi relativamente curto. Não acompanhamos o desfecho dos pacientes que deixaram de comparecer às consultas e/ou procedimentos em nosso hospital. Observamos as repercussões da pandemia da COVID-19 na prática assistencial de um hospital terciário como um todo, mas não caracterizamos as patologias que levaram os pacientes ao hospital.

## Conclusão

A pandemia da COVID-19 provocou importante redução no número de consultas nos ambulatórios de cardiologia, oncologia e demais especialidades no nosso hospital. Notamos também uma diminuição considerável no número de cirurgias cardíacas e nas sessões de quimioterapia e de radioterapia nas semanas iniciais da pandemia. A procura por atendimento no PS cardiológico, assim como o número de internações na UTI e enfermaria cardiológicas, também reduziu, gerando preocupação acerca da evolução e prognóstico destes pacientes portadores de outras patologias, que não a COVID-19, nestes tempos de pandemia.
