# Supplementary material for: Repercussões da Pandemia de COVID-19 na Prática Assistencial de um Hospital Terciário
Source: Arq Bras Cardiol. 2020 Sep 17;115(5):862–70. [Article in Portuguese] doi: 10.36660/abc.20200436 (PMC8452210; doi:10.36660/abc.20200436)
Supplement: Translation

# Translation: Repercussions of the COVID-19 Pandemic on the Care Practices of a Tertiary Hospital

**Authors:** André Luiz Cerqueira Almeida, Thyago Monteiro do Espírito Santo, Maurício Silva Santana Mello, Alexandre Viana Cedro, Nilson Lima Lopes, Ana Paloma Martins Rocha Ribeiro, João Gustavo Cerqueira Mota, Rodrigo Serapião Mendes, Paulo André Abreu Almeida, Murilo Araújo Ferreira, Diego Moreira Arruda, Adriana Aguiar Pepe Santos, Vinícius Guedes Rios, Maria Rosa Nascimento Dantas, Viviane Almeida Silva, Marcos Gomes da Silva, Patrick Harrison Santana Sampaio, André Raimundo Guimarães, Edval Gomes Santos

**Affiliations:** 1 Brazil Santa Casa de Misericórdia de Feira de Santana, Feira de Santana, BA - Brazil; 2 Brazil Escola de Ecocardiografia da Bahia, Feira de Santana, BA - Brazil; 3 Brazil Instituto Nobre de Cardiologia, Feira de Santana, BA - Brazil; 4 Brazil Universidade Estadual de Feira de Santana, Feira de Santana, BA - Brazil; 5 Brazil UNIFACS Curso de Medicina, Salvador, BA - Brazil

**Keywords:** COVID-19, Pandemics, Coronavirus, Betacoronavirus, Oncology, Hospitalization, Emergency Medical services

## Abstract

**Background:**

We still do not have information regarding the impact of the COVID-19 pandemic on medical care activity in Brazil.

**Objective:**

To describe the repercussions of the COVID-19 pandemic on the care routine of a tertiary hospital, which is a regional reference in cardiology and oncology.

**Methods:**

Cross-sectional cohort study. We conducted a survey of medical visits from March 23, 2020 (when local commerce was closed) to April 23, 2020 (P20), in comparison with the same period in 2019 (P19).

**Results:**

We found decreases in the number of cardiology consultations, exercise tests, Holter, ambulatory blood pressure monitoring, electrocardiogram, and echocardiogram (90%, 84%, 94%, 92%, 94%, and 81%, respectively). In relation to cardiac surgery and cardiac catheterization, there were 48% and 60% decreases, respectively. There was an increase in the number of percutaneous transluminal coronary angioplasties (33%) and definitive pacemaker implantations (29%). There were 97 admissions to the ICU during P19, in contrast with 78 during P20, a 20% decrease. Visits to the cardiac emergency room (45%) and admissions to the cardiology ward (36%) also decreased. The decrease in oncology consultations was 30%. Chemotherapy sessions decreased from 1,944 to 1,066 (45%), and radiotherapy sessions decreased by 19%.

**Conclusion:**

COVID-19 has led to a considerable decrease in the number of consultations in outpatient clinics for cardiology, oncology, and other specialties. There was a concerning decrease in the number of cardiac surgeries, chemotherapy sessions, and radiotherapy sessions during the initial weeks of the pandemic. The number of people seeking care in the cardiac emergency room and the number of admissions to the cardiology ward and ICU also decreased, generating concern regarding the evolution and prognosis of these patients with pathologies other than COVID-19 during this pandemic time. (Arq Bras Cardiol. 2020; [online].ahead print, PP.0-0)

## Introduction

In December 2019, the first cases of individuals infected with the new coronavirus (SARS-COV 2) were reported in Wuhan, China; the virus rapidly spread throughout the country, leading to a large number of deaths and hospitalizations.^1^ Within a short period of time, coronavirus disease 2019 (COVID-19) went beyond the limits of China, reaching other countries in Asia, Europe, and the Americas. By the middle of 2020, approximately 8.5 million people had tested positive for the new coronavirus in more than 187 countries and 200 territories, with 960,000 positive tests in Brazil. During this time, there were close to 450,000 deaths in the world attributed to COVID-19 (47,000 in Brazil).^2^ The first case of COVID-19 in Brazil was confirmed in São Paulo, on February 26, 2020.^3^ On March 6, 2020, the Secretary of Health of the State of Bahia confirmed the diagnosis of the first case of COVID-19 in Bahia, specifically, in the city of Feira de Santana. The patient was a 34-year-old woman, who had returned from Italy on February 25, having visited Milan and Rome, where contamination occurred. On March 11, 2020, the World Health Organization classified COVID-19 as a pandemic, and, on March 20, 2020, the Brazilian Ministry of Health declared community transmission of the disease throughout the national territory.^4^ This means that the virus was already circulating throughout the country. On the same date, the local public administrator of Feira de Santana issued a decree closing all wholesale and retail trade in the municipality, starting on March 23, 2020.

Scientific investigations are underway worldwide, and, as this is still a new incident, many efforts are being made to support health professionals and administrators. In Brazil in particular, any and all health conditions need to take into consideration the size of the country and its regional differences. During a pandemic like this one, the tripartite management structure of the Brazilian Unified Health System (SUS, acronym in Portuguese) becomes even more important, given that decisions must be shared between federal, state, and municipal governments.

During the pandemic, some authors have reported changes in the structures of healthcare systems in Europe and the USA, with an important decrease in the number of medical visits and procedures that are associated with COVID-19, including those of high complexity.^5 - 12^ As a collateral effect, these changes may lead to delayed diagnosis and/or therapy, with a consequent increase in the risk of decompensation of chronic diseases.

We still do not have information regarding the impact of the pandemic and subsequent government actions on medical care activity in Brazil. The objective of this study was to describe the repercussions of the COVID-19 pandemic on the number of consultations, tests, hospitalizations, and medical procedures carried out in a tertiary hospital, which is a regional reference for cardiology and oncology.

## Methods

This was a cross-sectional cohort study carried out in a tertiary hospital with 110 ward beds and 12 ICU beds. It is a regional reference unit in cardiology (cardiac surgery, catheterization, angioplasty, implantable electronic devices, echocardiogram, and cardiac emergency room [ER]) and oncology (chemotherapy, radiotherapy, and oncology surgery), and it provides care to both the SUS and the supplementary health system. In order to analyze the repercussions of the COVID-19 pandemic on the hospital’s care practices, a survey was carried out regarding the number of visits in the various sectors of the unit during the period from March 23, 2020 (the date when local commerce was closed) to April 23, 2020 (P20), in comparison with the services provided during the same period in 2019 (P19). This was a convenience sample, including all patients with data available from electronic records during the periods mentioned above. Data were collected on care practices and admission to the following sectors of the hospital: ICU (mainly cardiology), cardiac surgery, non-cardiac surgery, cardiac catheterization, percutaneous transluminal coronary angioplasty (PTCA), pacemaker implantation, cardiology consultations, echocardiography, ambulatory blood pressure monitoring (ABPM), Holter, electrocardiogram, exercise test, cardiac ER, admission to the cardiology unit, clinical laboratory analysis, oncology consultations, chemotherapy sessions, radiotherapy sessions, ultrasound, computerized tomography, endoscopy, colonoscopy, and rectosigmoidoscopy.

### Statistical Analysis

We carried out descriptive analysis of the data obtained in the sample. Nominal or categorical variables were described by their absolute values. The differences in events observed between the two study periods were described as absolute and relative ratios. Data analysis and graph construction were performed with the aid of Excel®, Microsoft 365®.

### Ethical Aspects

This study received approval from the Research Ethics Committee of the State University of Feira de Santana, under protocol number CAAE: 31056220.0.0000.0053. All of the procedures involved in this study are in accordance with the 1975 Declaration of Helsinki, revised in 2013. The survey was conducted by means of direct research of hospital records, following express authorization from the hospital. The researcher responsible signed an agreement form regarding use of data from hospital records.

## Results

During P19, there were 379 consultations at the cardiology outpatient clinic; this number decreased to 38 during P20, representing a 90% drop ( Figure 1 ). Decreases were also observed in the number of exercise tests (84%), Holter (94%), ABPM (92%), and electrocardiogram (94%). In relation to echocardiogram, there were 509 fewer exams during P20, corresponding to an 81% decrease ( Figure 1 ). This number comprised 470 outpatient echocardiograms (88%) and 39 exams carried out in patients who were hospitalized (41%).

**Figure 1:** – Number of cardiology consultations and exams. ABPM: ambulatory blood pressure monitoring; ECG: electrocardiogram; Echo: echocardiogram; ET: exercise test

In relation to cardiac surgery and cardiac catheterization, there were 48% and 60% decreases, respectively ( Figure 2 ).

**Figure 2:** – Movement in the interventional cardiology sector. CC: cardiac catheterization; PM: pacemaker; PTCA: percutaneous transluminal coronary angioplasty

There was a 33% increase in the number of PCTA and a 29% increase in definitive pacemaker implantations ( Figure 2 ).

During P19, 97 patients were admitted to the ICU, in comparison to 78 during P20, representing a 20% decrease. A decrease was also observed in the number of visits to the cardiac ER (45%) and in the number of admissions to the hospital’s cardiology ward (36%) ( Figure 3 ).

**Figure 3:** – Number of admissions to the cardiology unit. CW: cardiology ward; ER: emergency room; ICU: intensive care unit

The oncology sector also saw a considerable decrease in visits during the initial phase of the COVID-19 pandemic. Oncologists carried out 1,688 consultations during P19. This number dropped to 1,184 consultations during P20, a 30% decrease. The number of chemotherapy sessions dropped from 1,944 to 1,066, a 45% decrease. Radiotherapy sessions also decreased by 19% ( Figure 4 ).

**Figure 4:** – Movement in the oncology sector. CTS: chemotherapy sessions; RTS: radiotherapy sessions

There was a 42% decrease in the number of clinical analysis exams. This decrease comprises both outpatients and hospitalized patients; it was greater among the former ( Figure 5 ). There were 337 troponin assays in 2019, in contrast with only 59 in 2020, a 82% decrease.

**Figure 5:** – Movement of clinical laboratory analyses. Lab: laboratory exams

There was also a decrease in the number of the following procedures: endoscopy/colonoscopy/rectosigmoidoscopy (52%), ultrasound (94%), and computerized tomography (35%) ( Figure 6 ).

**Figure 6:** – Movement of imaging exams. Colono: colonoscopy; CT: computerized tomography; endo: upper digestive endoscopy; recto: rectosigmoidoscopy; US: ultrasound

Non-cardiac surgeries (general, oncology, head and neck, orthopedic, among others) saw a 40% reduction during the first month when social distancing was recommended due to the COVID-19 pandemic in the study region. The number of oncology surgeries related to urology decreased from 82 to 57 (30%). Non-cardiology and non-oncology consultations decreased by 92% ( Figure 7 ).

**Figure 7:** – Number of non-cardiac consultations and surgeries.

The hospital where data were collected is not a reference center for providing care for patients with COVID-19. Up to the moment when data collection was completed, no patients with COVID-19 had been admitted to the unit.

## Discussion

This study has shown that the COVID-19 pandemic led to a considerable decrease in the number of consultations at our hospital’s outpatient clinics in cardiology, oncology, and other specialties. Furthermore, we observed a concerning decrease in the number of cardiac surgeries, chemotherapy sessions, and radiotherapy sessions during the initial weeks of the pandemic. These decreases can lead to disastrous consequences for the patients who need these treatments. The number of people seeking care in the cardiac ER and the number of admissions to the cardiology ward and ICU also saw an important decline, leaving cardiologists and other healthcare professionals with a concerning question during this initial moment, namely: Where are patients with cardiac complications, whether or not they are associated with the COVID-19 pandemic, going?

We observed a 45% decrease in the number of visits to the cardiac ER in our hospital, with a 82% decrease in troponin assays. This was accompanied by 20% and 36% decreases in the rates of admission to the cardiology ICU and the cardiology ward, respectively. Similarly, Metzler B et al.^10^ demonstrated a 39.4% decrease in the admission of patients with acute coronary syndrome during the first month of the COVID-19 outbreak in Austria. In the USA, there was a 38% drop in coronary angiography in cases of acute ST-elevation myocardial infarction (STEMI) during the first month of the pandemic.^8^ In our study sample, we observed a 60% decrease in the number of cardiac catheterization procedures and a 48% decrease in the number of cardiac surgeries. In Spain, during the first week of quarantine due to COVID-19, researchers observed a 56% decrease in diagnostic cardiovascular procedures, a 48% decrease in coronary therapy procedures, an 81% decrease in structural therapy procedures (transcatheter aortic valve implantation, patent ductus arteriosus closure, and interatrial communication closure), and a 40% decrease in cases treated for STEMI.^8^ The decrease that we observed in individuals seeking medical attention for cardiac reasons during the pandemic, which has also been seen in other countries, goes against what we usually see during periods of tragedy. It is known that there is a considerable increase in the incidence of acute myocardial infarction and stroke following earthquakes and tsunamis.^13 , 14^ Perhaps, as a result of this decrease in spontaneous demand for medical care in cases that not related to COVID-19, the region of Lombardy, Italy, showed a 58% increase in the occurrence of cardiac arrest outside the hospital during the period that included the first 40 days of the COVID-19 outbreak.^12^ This finding showed a strong association with the cumulative incidence of COVID-19 in the region studied. Similarly, data published on Angioplasty.Org report an 800% increase in the incidence of sudden deaths occurring at home in the city of New York when it was the epicenter of the pandemic.^15^ Many of these patients may have avoided going to the hospital due to fear of becoming infected with COVID-19.^16^

In contrast with the data obtained in Spain,^8^ we observed a 33% increase in PCTA. We attribute this increase to the greater availability of beds in our hospital’s ICU during the initial phase of the COVID-19 pandemic. This made it possible for us to carry out the procedure and transfer patients to the ICU for observation, which is a different scenario from the pre-COVID phase, when the ICU, invariably, had no beds available.

The decrease which we observed in the number of oncology consultations (30%), chemotherapy sessions (45%), and radiotherapy sessions (19%) is a different case. A recent study, carried out in England and North Ireland, observed that the majority of patients with cancer or suspicion of cancer were not accessing health services during the COVID-19 pandemic.^17^ As a consequence, it is estimated that this COVID-19 outbreak has the potential to increase mortality by approximately 20%, over the coming 12 months, in patients with recent diagnosis of cancer, in England alone.^17^ Moreover, patients with cancer have an almost four-fold risk of presenting severe complications secondary to COVID-19, when compared to individuals without cancer.^18^ Therefore, monitoring of patients in oncology should be doubled, rather than decreased, during the pandemic. The challenge in identifying and treating complications associated with some chemotherapy drugs, such as severe myocarditis and/or pneumonitis, is another issue which specialists need to face during the COVID-19 pandemic.^19^

Our results are of great assistance to patients, healthcare professionals, and hospital managers. They shine a light on a serious problem, which is occurring in parallel to the COVID-19 pandemic, namely, a considerable decrease in the number of consultations, cardiac and oncological surgeries, visits to the cardiac ER, cardiac catheterization, chemotherapy and radiotherapy sessions, laboratory tests, and other medical procedures that are important and necessary for patients who do not have COVID-19. Decreased access to medical care is associated with a decline in the population’s health status.^20^ Delayed diagnosis and treatment of myocardial infarction or stroke increase the risk of death.^21^ Interrupting the use of anti-hypertensive medication, even for short periods of time, may lead to severe cardiovascular complications.^22^ Withdrawal of statins increases rates of events in patients with acute coronary syndromes.^22^ Undiagnosed decompensation of diabetes has severe consequences. Delayed performance of oncological surgery can lead to disease progression and worsened prognosis. The same applies to adjuvant or neoadjuvant chemotherapy sessions. The fact that patients are abstaining from going to the hospital does mean that other diseases have disappeared. By any means! They continue affecting patients, but patients are not seeking medical care to the appropriate extent. The question is: Why? Among other reasons, they are probably afraid of going to the hospital on account of the risk of contracting COVID-19. It is also possible that some patients with cardiovascular problems are seeking medical attention, but the symptoms they present may be confused with those of COVID-19. Express recommendations for people to stay home, in addition to restrictions on traffic and movement, also contribute to the scenario that we have detected in our hospital. It would not be an exaggeration to say that these individuals have also become victims of COVID-19, even without having contracted the disease.

Regardless of the cause, the results of our study reflect what is happening in several other places. These facts have the potential to generate a worrying collateral effect, namely, a substantial increase in morbidity and mortality, both short and medium term, due to causes other than the infection caused by SARS-COV 2.^16^ This would be the other side of the COVID-19 tragedy. It is possible that, soon, we will face an ascending, post-pandemic curve composed of patients with serious complications secondary to pathologies that could have been adequately treated, had they been previously attended at a time which would have, theoretically, been more favorable.

Our results open perspectives for other researchers to investigate the real outcomes of patients who are not attending elective medical consultations, chemotherapy sessions, radiotherapy sessions, and the cardiac ER, as well as those who are not undergoing routine examinations and/or oncological or cardiac surgeries during the COVID-19 pandemic. This would complement our findings and bring important information to the medical community and to patients themselves.

There are some limitations to this study. It was a single center study, and the observation period was relatively short. We did not follow up the outcomes of patients who stopped coming to consultations and/or procedures in our hospital. We observed the repercussions of the COVID-19 pandemic on the care practice of a tertiary hospital as a whole, but we did not characterize the pathologies that brought patients to the hospital.

## Conclusion

The COVID-19 pandemic caused an important reduction in the number of consultations in the cardiology, oncology and other specialties outpatient clinics at our hospital. We also noticed a considerable decrease in the number of cardiac surgeries and in chemotherapy and radiotherapy sessions in the initial weeks of the pandemic. The demand for care in the cardiac ER, as well as the number of hospitalizations in the ICU and cardiology ward, also reduced, generating concern about the evolution and prognosis of these patients with other pathologies, other than COVID-19, in these pandemic times.
